# Arrhythmia onsets triggered by acute myocardial ischemia are not mediated by lysophosphoglycerides accumulation in ventricular myocardium

**DOI:** 10.1038/s41598-024-57047-5

**Published:** 2024-04-26

**Authors:** Jiawei Liu, Tingting Mai, Han Ren, Yafei Chang, Chao Li, Guoli Lv, Da Zheng, Xinbiao Liao, Yangeng Yu, Fu Zhang, Shuquan Zhao, Xiaoshan Liu, Shuiping Liu, Hu Zhao, Bin Luo, Chao Liu, Erwen Huang

**Affiliations:** 1 Faculty of Forensic Medicine, Guangdong Province Translational Forensic Medicine Engineering Technology Research Center, Zhongshan School of Medicine, Sun Yat-Sen University, Guangzhou, 510080 China; 2 Department of Clinical Medicine, Zhongshan School of Medicine, Sun Yat-Sen University, Guangzhou, 510080 China; 3 Guangzhou Forensic Science Institute, Guangzhou, 510030 China; 4 Key Laboratory of Forensic Pathology, Ministry of Public Security, Guangzhou, 510050 China; 5 National Anti-Drug Laboratory Guangdong Regional Center, Guangzhou, 510230 China

**Keywords:** Cardiovascular biology, Cardiology

## Abstract

Lysophosphoglycerides (LPLs) have been reported to accumulate in myocardium and serve as a cause of arrhythmias in acute myocardial ischemia. However, in this study we found that LPLs level in the ventricular myocardium was decreased by the onset of acute myocardial ischemia in vivo in rats. Decreasing of LPLs level in left ventricular myocardium, but not right, was observed within 26 min of left myocardial ischemia, regardless of whether arrhythmias were triggered. Lower LPLs level in the ventricular myocardium was also observed in aconitine-simulated ventricular fibrillation (*P* < 0.0001) and ouabain-simulated III^°^ atrioventricular block (*P* < 0.0001). Shot-lasting electric shock, e.g., ≤ 40 s, decreased LPLs level, while long-lasting, e.g., 5 min, increased it (fold change = 2.27, *P* = 0.0008). LPLs accumulation was observed in long-lasting myocardial ischemia, e.g., 4 h (fold change = 1.20, *P* = 0.0012), when caspase3 activity was elevated (*P* = 0.0012), indicating increased cell death, but not coincided with higher frequent arrhythmias. In postmortem human ventricular myocardium, differences of LPLs level in left ventricular myocardium was not observed among coronary artery disease- and other heart diseases-caused sudden death and non-heart disease caused death. LPLs level manifested a remarkable increasing from postmortem 12 h on in rats, thus abolishing the potential for serving as biomarkers of sudden cardiac death. Token together, in this study we found that LPLs in ventricular myocardium were initially decreased by the onset of ischemia, LPLs accumulation do not confer arrhythmogenesis during acute myocardial ischemia. It is necessary to reassess the roles of LPLs in myocardial infarction.

## Introduction

Sudden cardiac death (SCD) is responsible for a large proportion of mortality. Data from both clinical and forensic departments indicated an annual incidence of about 41.8–60 per 100,000 individuals^1,2^, which accounted for approximately 10% or even more of overall mortality^2–4^, and near 45–70% of sudden death^4–6^. A special type of SCD was termed sudden unexplained death (SUD). The proportion of SUD in overall increases with the decrease of age^3–7^. It has been well recognized that SUD, or at least a proportion of them, are caused by tachy- or brady-lethal ventricular arrhythmias led from non-structural disorders, e.g., ion channel diseases^7,8^ and concealed cardiomyopathies^9^. These disorders can not be discovered by autopsy, histopathological and imageological examinations, owing to the absence of morphological lesions. Although genetic factors of SUD have been well studied, the molecular mechanisms of the onset of ventricular arrhythmias remain still poorly understood. Discovering the molecular mechanisms and identifying relevant biomarkers of SUD attack will not only provide more direct proofs for forensic purpose, but also help to enhance the clinical intervention and to evaluate the resuscitation prognosis. Moreover, SCD caused by structural heart diseases, e.g., myocardial ischemia, usually undergo the same arrhythmias to SUD, suggesting the benefit of SUD knowledge for understanding and treating with structural SCD.

Two species of lysophosphoglycerides (LPLs), lysophosphatidylcholine (LPC) and lysophosphatidylethanolamine, have been reported to accumulate rapidly in ischemic myocardium in rabbit^10^, cat^11,12^, pig^13^, dog^14^ and rat hearts^15^. Based on analysis of the extracts from the effluents of anoxic isolated rabbit hearts, LPLs altered dog Purkinje fiber action potentials in vitro, at similar concentrations to the sum of overall concentrations in ischemic myocardium in situ^10,16^. Perfusion with a LPC-containing solution caused cardiac arrhythmia in hamster hearts^17^. Exogenous LPC of low concentration was shown to produce cardiac damage similar to that caused by ischemia^15,18^.

Multiple mechanisms under which LPLs induces arrhythmias and cardiac damage were reported. LPC modulated cardiac sodium channel^19–21^, enhanced I_Kr_ and I_Ks_ currents^22,23^, thus altered the action potential of cardiomyocytes. It modulated the serine phosphorylation of connexin43^24^, and reduced gap junctional coupling in cardiac cells^25^. It up-regulated T-type, or else other types of calcium channels and led to Ca^2+^-overloading in cardiomyocytes^26–28^. It also induced mitochondrial fission and in turn contributed to collagen production in cardiac fibroblasts^29^.

To discover lipid biomarkers of SCD, in this study we investigated lipidomic changes in arrhythmic and ischemic rat ventricular myocardia in vivo. We found the onset of ischemia decreased the level of main LPLs in the ventricular myocardium, inconsistent with the previous reports that LPLs accumulated in early ischemic myocaridum^10–15^. We further found long-lasting ischemia indeed increased LPLs level, but high level of LPLs did not increased the risk of arrhythmias in vivo, conflicting with the concept that accumulation of LPLs was a trigger of arrhythmias in myocardial ischemia^10–17^. We found that LPLs accumulation coincided with cell death, and the postmortem accumulation of LPLs limited them from serving as SCD biomarkers for forensic purpose.

## Materials and methods

### Reagents and instruments

Pentobarbital Sodium (Sigma, P3761); Aconitine (Wanwu standard technology limited company, CAS: 302-27-2); Ouabain (Shanghai Macklin biochemical technology limited company, CAS: 11018-89-6); Saline (Shanghai Baite medical supplies limited company, S1707084); PBS buffer; Acetonitrile (Thermo Fisher); Isopropanol (Thermo Fisher); Methanol (Thermo Fisher); Ammonium formate (Sigma, 70221).

Mouse Monitor TMS small animal physiological signs real-time monitor (INDUS); Q-exactive Plus mass spectrometer (Thermo Scientific); UHPLC Nexera LC-30A ultra-high performance liquid chromatography (Shimadzu); Low temperature high speed centrifuge (Eppendorf 5430R); The chromatographic column: Waters, Acquity UPLC CSH C18, 1.7 µm, 2.1 mm × 100 mm column.

### Establishment of drug-simulated SUD models in rat

Sprague–Dawley rats aged 7 weeks (body weight 184–219 g) from the Animal Experiment Center in Sun Yat-sen University (production license number: SCXK (Guangdong) 2004-0011. License number: SYXK (Guangdong) 2010-0107 were used in this study.

Rats were anesthetized by intraperitoneal injection of pentobarbital sodium solution (0.04 g/kg) and fixed on the operation table of a small animal physiological signs real-time monitor. In SUD groups, aconitine (0.05 g/L, 1 mL/rat) or ouabain (2.00 g/L, 1 mL/rat) dissolved in normal saline was injected via tail vein, to induce ventricular tachycardia and ventricular fibrillation or atrioventricular block respectively. Results of preliminary experiments showed that such drug doses caused most asystole in 15–60 min, mimicing the time description in the definition of sudden cardiac death (sacrificed in no longer than 1 h after the onset of heart attack). In control group, normal saline (1 mL/rat) was administrated via tail vein, and rats sacrificed by cervical dislocation in 30 min after saline injection.

### Establishment of acute myocardial ischemia models

Rats under the anaesthetic were fixed on the operating table on their back and barbered on the left thorax. Electrocardiogram electrodes were connected under the skin of the limbs, and the II lead electrocardiogram was recorded. The skin and muscle between the 3rd and 4th ribs of the left thorax were cut, interval between the ribs was spread. The thymus was pushed to upper right, the pericardium was cut to expose the heart and the roots of large vessels. The left coronary artery between the left auricle and the pulmonary conus was ligated using suture line no. 6-0. Acute myocardial ischemia was indicated to be induced successfully by that the myocardium perfused by the ligated artery became pale and the elevation of the ST segment in the electrocardiogram. Then hemostasis, exhausting the air from the thorax and suturing. In group of ischemia in the right myocardium, the right coronary artery between the right auricle and the pulmonary conus was ligated. In sham group, the operation was the same to that in the ischemia group but not knotting the suture line. The electrocardiogram was recorded until death.

### Electric shock assay

Rats under anaesthetic were fixed on the operating table on their back. The II lead electrocardiogram was recorded before electric shock. One end of the wire was contacted with the wrist of the left upper limb, the other end with the ankle of the right lower limb, or both ends were contacted with the ankle of the left and right lower limb respectively. Circuit with 220 V voltage was turned on for 10, 20, 40 s, 2 and 5 min. All of the rats were sacrificed by cervical dislocation as soon as at the end of mentioned electric shock time.

### Preparation of ventricular myocardium samples

After animals sacrificed by cervical dislocation or asystole, ventricular myocardia were separated immediately, washed with pre-cooled PBS buffer to get rid of blood, wiped dry and cut up on ice, and then stored −80 ℃. In the experiments of drugs- and electric shock-induced arrhythmias, the left and right ventricular myocardia were collected and analyzed together as an entirety, and in those of coronary artery occlusion the two parts of ventricular myocardia were harvested independently for disclosing the potential differences of LPLs levels between in ischemic and non-ischemic areas.

### Sample pre-treatment

For each experimental subject, a sample (40 mg) was mixed with 200 μL of water, then homogenized, combined with pre-cooled methanol (240 μL) and MTBE (800 μL) successively. Each combination was followed by vortex mixing. The specimen was ultrasonicated in ice water for 20 min, kept still at room temperature for 30 min, centrifuged at 8000 g 10 ℃ for 15 min. Organic phase in the specimen was isolated and dried by blowing nitrogen, then re-dissolved with 400 μL of isopropyl alcohol, centrifuged at 8000 g 10 ℃ for 15 min. Supernatant was isolated for LC–MS analysis.

### LC–MS/MS analysis

Samples were treated using UHPLC Nexera LC-30A system. Briefly, the procedure and condition were set as: column temperature 45 ℃, flowing rate 300 μL/min, injection volume 2μL, mobile phase A: ammonium formate acetonitrile solution (10 mM, acetonitrile:water = 6:4, v/v), mobile phase B: ammonium formate acetonitrile isopropanol solution (10 mM, acetonitrile:isopropanol = 1:9, v/v). Gradient elution procedure: mobile phase B was maintained at 30% for 1 min, changed linearly from 30 to 100% for 24 min, and maintained at 30% again for 5 min. The temperature of automatic sampler was set at 10 ℃, during analysis. Samples were analyzed in random order, without intermission between each other. To evaluate the system stability and data reliability, one Quality control (QC) sample was added for every 8 experimental samples. Samples after the UHPLC analysis were tested using Q Exactive plus mass spectrometer (Thermo Scientific™), under electrospray ionization positive and negative ion modes. Testing condition was set as: (positive) spray voltage 3.0 kV, heater temperature 300 °C, capillary temperature 350 °C, sheath gas flow rate 45arb, aux gas flow rate 15arb, sweep gas flow rate 1arb, S-Lens RF Level 50%. MS1 scan ranges 200–1800; (negative) spray voltage 2.5 kV, heater temperature 300 °C, capillary temperature 350 °C, sheath gas flow rate 45arb, aux gas flow rate 15arb, sweep gas flow rate 1arb, S-Lens RF level 60%. MS1 scan ranges 250–1800. Ten fragments maps (MS2 scan, HCD) were collected after each full scan for calculating mass charge ratio. The resolution of MS1 at *m/z* 200 is 70,000, and that of MS2 at *m/z* 200 is 17,500.

### Human specimens

Human left ventricular myocardia were isolated when autopsy and stored −80 ℃ for the LC–MS/MS test. Sixty-six specimens were enrolled in this study, including 16 SCD caused by coronary artery disease, four SCD caused by non-coronary artery disease, and 46 non-SCD. The cause of death of the dead was determined by autopsy, histological examination and forensic toxicology test. For cases possible anaphylactic shock, IgE testing and toluidine blue staining were additionally performed. Genders, age, diseases, causes of death and postmortem interval before autopsy were recorded. Obsolete infarct areas in the myocardia were excluded from analyses.

### Data analysis

Row data collecting and lipids identification were performed with software LipidSearch v4.1 (Thermo Scientific™). Main parameters in the analysis were set as: precursor tolerance 5 ppm, product tolerance 5 ppm, product ion threshold 5%. Data with relative standard deviation > 30% or missing value > 50% were excluded from further analysis. Pareto-scaling, principal component analysis, partial least squares discriminant analysis and orthogonal partial least squares discriminant analysis were carried out using software SIMCA-p v14.1. Hierarchical clustering analysis was performed with R language v3.6.1.

### Ethics approval

The animal experiments conformed to the Guide for the Care and Use of Laboratory Animals and were approved by the Ethics Committee of ZSSOM on Laboratory Animal Care and Use in Sun Yat-sen University (Approval Number: SYSU-IACUC-2020-B0081). All methods used in animal experiments were carried out in accordance with relevant guidelines, and handled according to standard use protocols and animal welfare regulations. All animal studies reported were performed in accordance with ARRIVE guidelines (https://arriveguidelines.org).

The collection and use of human specimens were approved by informed consents from the bereaved families, and reviewed and approved by the Medical Ethics Committee of Zhongshan School of Medicine, Sun Yat-sen University (Approval number: Zhongshan Medicine Medical Ethics [2020] No. 050). The investigations were carried out following the Declaration of Helsinki.

## Results

### Animal models of SCD

In aconitine group, ventricular tachycardia was observed in about 2 min after drug administration. All of the rats underwent ventricular premature beat, ventricular tachycardia and ventricular fibrillation. Asystole occurred in 17–30 min after aconitine injections, with a mean time of 22.7 ± 4.8 min. In ouabain group, atrioventricular block appeared in 1.5 min after drug administration and lasted for a time ranging from 20 to 50 min (mean 35.1 ± 9.7 min), followed by asystole. In saline solution group, no abnormal ventricular rhythm was observed. In group of acute myocardial ischemia, the onset time of arrhythmias, including tachycardia and bradycardia, could be seen from shorter than one minute to longer than 10 min. In some animals, III^°^ atrioventricular block or ventricular fibrillation was followed by asystole (grouped as SCD), and in others the electrocardiogram was recovered to normal except for ST segment elevation. An example of electrocardiogram was referred in Supplementary Fig. S1.

### Alteration of lipidomic profiles in the ventricular myocardia of drug-simulated SUD

**Figure 1:**
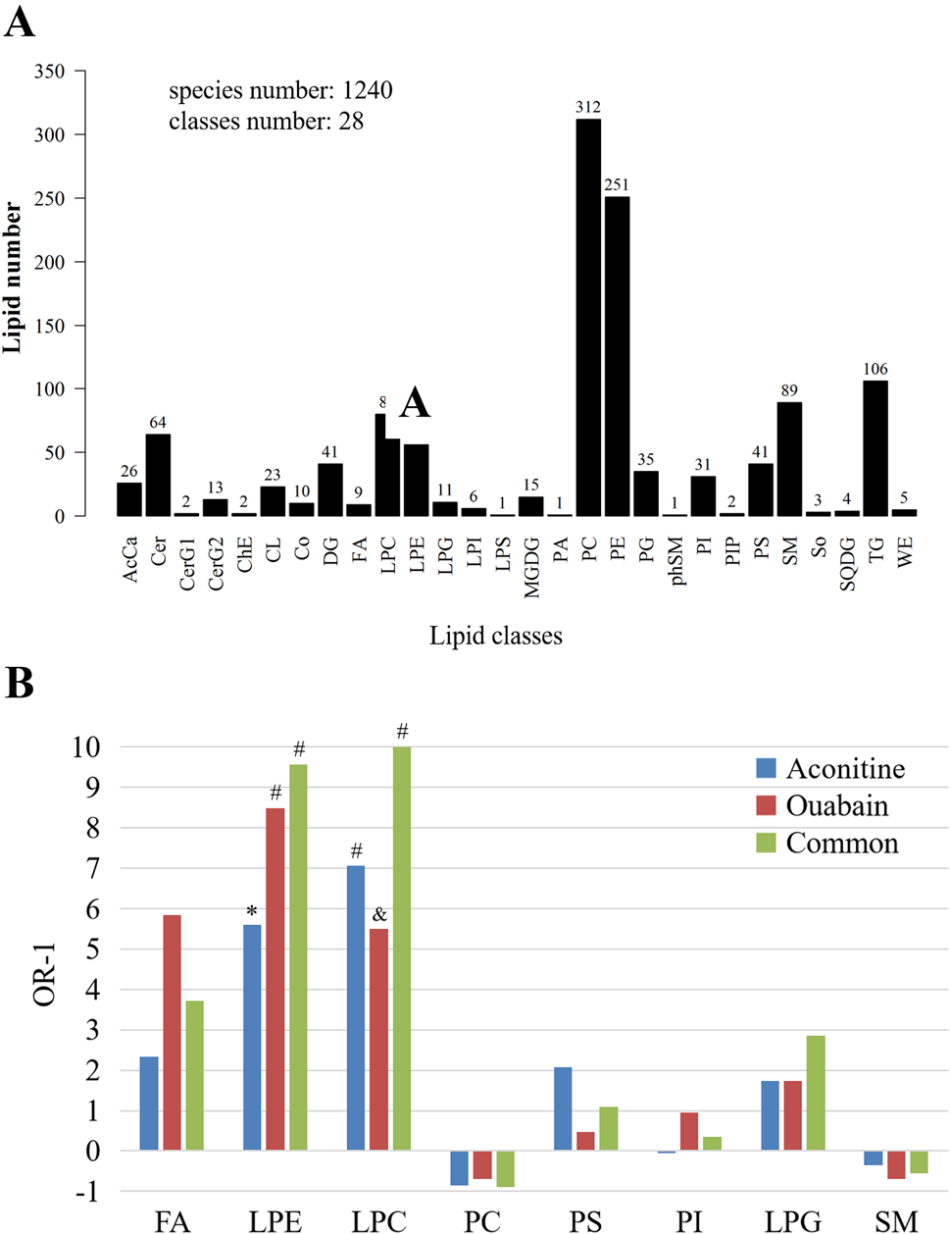
Alteration of lipid profiles in ventricular myocardia in aconitine- and ouabain-simulated SUD models. (**A**) Numbers of identified lipid species and classification of them. (**B**) Odds ratios of lipid classes in the differential lipid ions to them in the whole identified lipidomic pool. N = 10 in each of the groups ^*^P = 0.000026, ^&^*P* = 0.000003, ^#^*P* ≤ 0.000001.

Quality of lipidomic tests was controlled by QC sample spectrogram comparison and PCA. Responding intensity and retention time in the base peak spectrograms of QC samples overlapped well (Supplementary Fig. S2A, B), suggesting the tests were repeatable and stable. Similar results were observed in PCA (Fig. S2C). QC samples in PCA diagram were closely clustered together and located in the middle of each group. A total of 1240 lipid ions (including 787 cations and 453 anions) classified in 28 classes were identified in each of the groups (Fig. 1A). PCA and OPLS-DA based on lipid profiles revealed that lipidomes in ventricular myocardia in both aconitine and ouabain induced SUD models altered considerably, compared to saline control group (Supplementary Fig. S3).

We screened differential molecules in the identified lipids on the criteria of *P* < 0.05 in *t* test and Variable Importance for the Projection > 1 in the OPLS-DA. As a result, forty-two lipid ions were screened out in both of the SUD models, respectively (Supplementary Fig. S4), thirty of which were common in the models (Table 1). Among the 30 lipid ions, there were nine pairs of lipids with the same fatty acid chain but different ionic groups. Hierarchical clustering analysis showed that levels of the 30 lipid ions differentiated SUD from control clearly (Supplementary Fig. S5).

**Table 1:** Differential lipid ions common in aconitine- and ouabain-simulated simulated SUD models.

Lipid ion	Aconitine vs control				Ouabain vs control			
	FC	VIP	*P*-value	FDR	FC	VIP	*P*-value	FDR
LPC(16:0) + H	0.57	5.38	4.67E−03	0.075	0.62	4.33	1.52E−02	0.112
LPC(16:0) + HCOO	0.58	5.43	1.30E−03	0.048	0.62	4.81	6.59E−03	0.077
LPC(18:0) + H	0.43	10.20	6.43E−05	0.011	0.47	9.09	1.07E−04	0.013
LPC(18:0) + HCOO	0.58	7.93	1.61E−05	0.007	0.58	7.02	3.33E−04	0.020
LPC(18:1) + H^a^	0.54	2.20	4.34E−04	0.031	0.57	1.93	1.37E−03	0.042
LPC(18:1) + H^b^	0.42	2.89	2.25E−03	0.055	0.47	2.51	5.13E−03	0.069
LPC(18:1) + HCOO	0.40	2.80	5.94E−03	0.081	0.48	2.24	2.15E−02	0.142
LPC(18:2) + HCOO	0.48	3.47	2.66E−03	0.059	0.57	2.60	1.65E−02	0.118
LPC(18:3) + H	0.61	1.17	2.30E−03	0.055	0.65	1.02	6.93E−03	0.077
LPC(20:4) + H	0.72	2.57	1.99E−03	0.054	0.60	3.12	1.63E−04	0.016
LPC(20:4) + HCOO	0.65	2.94	7.51E−04	0.044	0.63	2.93	1.01E−03	0.036
LPC(22:6) + H	0.59	2.42	4.40E−04	0.031	0.58	2.28	2.13E−03	0.050
LPC(22:6) + HCOO	0.66	2.08	3.19E−03	0.062	0.51	2.64	9.60E−05	0.013
LPE(16:0) + H	0.66	1.38	7.83E−03	0.095	0.53	1.67	7.49E−04	0.031
LPE(16:0) − H	0.63	3.11	8.10E−03	0.095	0.51	3.52	1.59E−03	0.044
LPE(18:0) + H	0.67	2.57	8.53E−03	0.097	0.64	2.49	2.46E−03	0.053
LPE(18:0) − H^c^	0.63	6.86	8.15E−03	0.095	0.52	7.69	1.66E−03	0.044
LPE(18:0) − H^d^	0.45	1.10	1.80E−02	0.165	0.37	1.23	5.33E−03	0.069
LPE(18:2) + H	0.61	1.15	2.19E−02	0.183	0.51	1.23	5.23E−03	0.069
LPE(20:4) − H	0.75	1.99	5.03E−03	0.080	0.68	2.21	3.21E−03	0.058
LPE(22:5) − H	0.64	1.05	4.42E−03	0.075	0.57	1.02	2.96E−03	0.058
LPE(22:6) + H	0.77	1.65	8.07E−03	0.095	0.66	2.06	2.09E−03	0.050
LPE(22:6) − H	0.77	3.51	4.17E−02	0.251	0.62	4.57	2.34E−03	0.052
LPG(16:0) − H	0.51	1.24	3.72E−02	0.239	0.47	1.21	2.57E−02	0.157
PC(18:1/20:4) + HCOO	1.77	1.89	1.44E−02	0.138	1.58	1.51	3.42E−02	0.184
PS(18:0/22:6) + H	2.16	1.75	9.25E−03	0.102	1.52	1.23	1.61E−03	0.044
PS(18:0/22:6) − H	2.14	3.75	3.77E−03	0.070	1.46	2.57	5.08E−05	0.009
PI(34:1) − H	1.82	2.56	9.71E−03	0.106	1.29	1.54	5.19E−03	0.069
FA(20:4) − H	0.69	1.92	4.00E−03	0.070	0.63	2.10	1.47E−03	0.043
SM(d40:1) + H	0.75	2.83	4.19E−06	0.003	0.84	1.86	2.01E−04	0.017

*FC* fold change, *VIP* variable importance for the projection, *FDR* false discovery rate, ^a^RT (retention time) = 3.10 min.

^b^RT = 3.31 min.

^c^RT = 4.73 min.

^d^RT = 5.8 min.

To our attention, as many as 28 out of the 30 lipid ions were glycerophospholipids, including 13 LPCs, 10 LPEs, 1 LPG, 1 PC, 2 PSs and 1 PI (Table 1). Odds ratios of LPC and LPE proportions in the differential lipid ions to that in the whole identified pool were no less than 6.50 (*P* ≤ 0.000026) in a single model, and in the common pool their odds ratios reached to 11.09 (*P* < 0.000001) and 10.57 (*P* = 0.000001), respectively (Fig. 1B).

### LPLs level in the ventricular myocardia decreased in drug-simulated SUD and electric shock.

Another attracting point was that all of the differential LPLs were down-regulated (Table 1), and this was true in almost all of the ions that were not statistically different between SUD and control groups (Supplementary Appendix sheet 1), suggesting the alteration of some shared metabolic pathways. A total of 154 LPL ions (80 LPCs, 56 LPEs, 11 LPGs, 6 LPIs and 1 LPS) were detected, and content sum of the statistically different ions occupied more than 87% of that of all of the detected LPL ions, (Supplementary Appendix sheet 1). Content sums of 7 LPLs (LPC16:0, LPC18:0, LPC18:1, LPE16:0, LPE18:0, LPE18:1 and LPG16:0) in the two ion pools with or without statistical difference accounted for 76.32% and 72.98%, respectively (Supplementary Appendix sheet 2 and 3), similar to that in porcine hearts reported previously^13^. Thus the 7 LPLs were used to represent all of the LPL ions in the follow-up study. As shown in Supplementary Fig. S6, decrease of the 7 LPLs in the ventricular myocardia was repeated in both of the drug-simulated SUD models.

**Figure 2:**
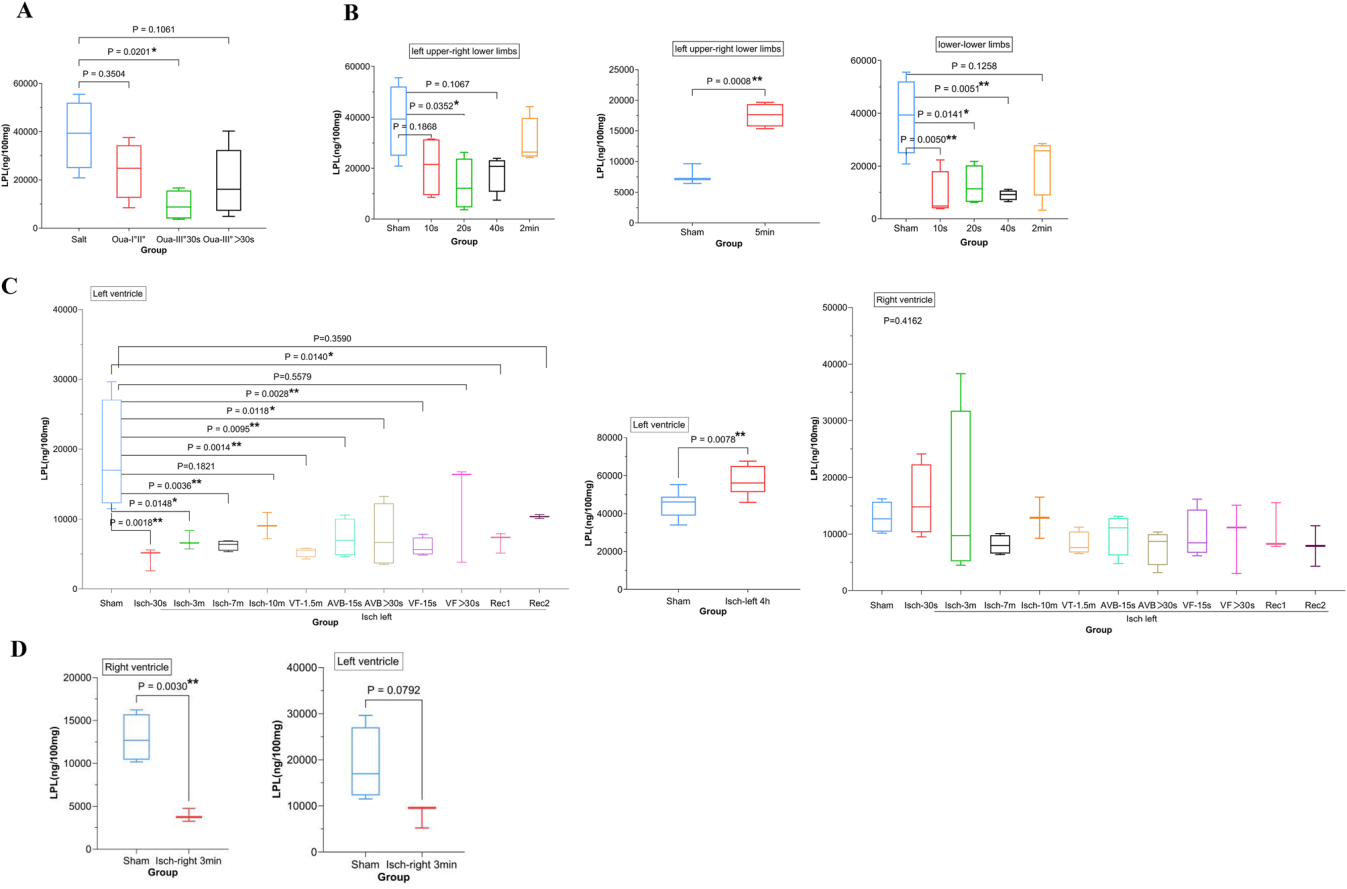
(**A**) Time-course changes of LPLs levels in whole-ventricular myocardia along with the development of arrhythmias in ouabain-simulated SUD. Salt: rats sacrificed by cervical dislocation in 30 min after saline injection (n = 5). Oua-I°II°: ouabain-induced I°–II° atrioventricular block lasting 30 min and sacrificed by cervical dislocation (n = 5). Oua-III°30 s: ouabain-induced III° atrioventricular block lasting 30 s and sacrificed by cervical dislocation (n = 5). Oua-III° > 30 s: ouabain-induced III° atrioventricular block lasting longer than 30 s before asystole (n = 7). (**B**) Time-course changes of LPLs levels in whole-ventricular myocardia with electric shock via left upper-right lower limbs current path or lower-lower limbs current path (n = 5 in each group). (**C**) LPLs levels in ventricular myocardia in left coronary artery occlusion-induced myocardial ischemia (n = 4–7 in each group). (**D**) LPLs levels in ventricular myocardia in right coronary artery occlusion-induced myocardial ischemia for 3 min without arrhythmias (n = 5 in each group). *Isch* ischemia, *VT* ventricular tachycardia, *AVB:* *III*° atrioventricular block, *VF* ventricular fibrillation, *Rec* *1* the ECG recovered to normal following transient I°–II° atrioventricular block, *Rec* *2* the ECG recovered to normal following transient III° atrioventricular block.

We further investigated a time-course change of LPLs along with the development of arrhythmias in the ouabain-simulated SUD. As shown in Fig. 2A and Supplementary Table S1, LPLs level in the whole ventricular myocardium with I^°-^II^°^ atrioventricular block decreased in a certain degree (fold change = 0.61, *P* = 0.35), and decreased further in that with III^°^ atrioventricular block lasting 30 s (fold change = 0.24, *P* = 0.02), compared to salt group. In group of III^°^ atrioventricular block lasting longer than 30 s (arrhythmia lasting from 30 s to 26 min before asystole), LPLs level was still lower than that in salt group, although not statistically significant (fold change = 0.39, *P* = 0.11).

Electric current through the heart induces ventricular fibrillation. Here, we tested the time-course changes of LPLs levels in whole-ventricular myocardia in electric shock. In left upper-right lower limbs current path, LPLs levels in the ventricular myocardium decreased mildly in group of 10-s electric shock (fold change = 0.55, *P* = 0.19) and decreased further in group of 20-s electric shock (fold change = 0.36, *P* = 0.04), compared to that in sham group. When extending the time of electric shock, LPLs levels made a U-turn, and surpassed the level in sham group at no longer than 5 min (FC = 2.27, *P* = 0.0008). In lower-lower limbs current path, lower LPLs levels were observed again in electric shock for time no longer than 2 min (Fig. 2B, Supplementary Table S2).

These results suggested that arrhythmia-triggering factors, e.g., drugs and electric current, can decrease LPLs levels in the whole-ventricular myocardium. And if these factors last long enough, the LPLs levels will make a U-turn.

### LPLs do not confer arrhythmogenesis during acute myocardial infarction

We studied whether this alteration would repeat in acute myocardial ischemia. As shown in Fig. 2C left and Supplementary Table S3, left myocardial ischemia free of arrhythmias for 0.5, 3 and 7 min decreased the LPLs levels in left ventricular myocardia significantly (fold change ≤ 0.33, *P* ≤ 0.0036). As the time of arrhythmia-free ischemia extended to 10 min, lower LPLs level in left ventricular myocardia than that in sham group was still maintained (fold change = 0.48, *P* = 0.18). In groups of left myocardial ischemia with 1.5-min ventricular tachycardia, 15- and > 30-s III° atrioventricular block, and 15-s ventricular fibrillation, similar results were obtained (fold change ≤ 0.40, *P* ≤ 0.01, Fig. 2C left and Supplementary Table S3). In these groups, ischemia lasted no longer than 8.4 min and arrhythmias lasted no longer than 6.2 min (Supplementary Table S3). In a group that ischemia lasted 9 min with > 30-s ventricular fibrillation (5.3 min for all types of arrhythmias), lower LPLs level was still observed, although without statistic significancy (fold change = 0.66, *P* = 0.56). In two groups of 26-min ischemia, both of which experienced 0.7 min of transient arrhythmia, lower LPLs level continued, regardless of I°-II° atrioventricular block (fold change = 0.36, *P* = 0.014) or III° (fold change = 0.55, *P* = 0.36), although not statistically significant in the latter. When ischemia time was extended further to 4 h, when high frequent ventricular arrhythmia storms had gone, the LPLs level in ischemia group surpassed that in the sham group (fold change = 1.20, *P* = 0.0078, Fig. 2C middle and Supplementary Table S3). Unlike in left ventricular myocardia, LPLs levels in right ventricular myocardia were not significantly altered in any of the groups of left myocardial ischemia lasting no longer than 26 min (Fig. 2C right and Supplementary Table S3). In the group of right myocardial ischemia for 3 min without arrhythmias, remarkable decreasing of LPLs levels was observed in right ventricular myocardia (fold change = 0.30, *P* = 0.003), but not in left (fold change = 0.43, *P* = 0.079, Fig. 2D and Supplementary Table S3).

Taken together, these results indicated that (1) the onset of acute myocardial ischemia decreased LPLs level in the ischemic myocardium, (2) ischemia-induced arrhythmias were not responsible for the down-regulation of LPLs. (3) long-lasting rather than the onset of ischemia caused LPLs accumulation in the ventricular myocardium, (4) ischemia-induced arrhythmias were independent of LPLs accumulation. These observations were inconsistent with the previous conclusion that LPLs accumulated in ischemic myocardium and induced arrhythmia early after the onset of acute myocardial ischemia^10–15^.

### LPLs accumulated in dead tissues and could not serve as biomarkers of SCD

**Figure 3:**
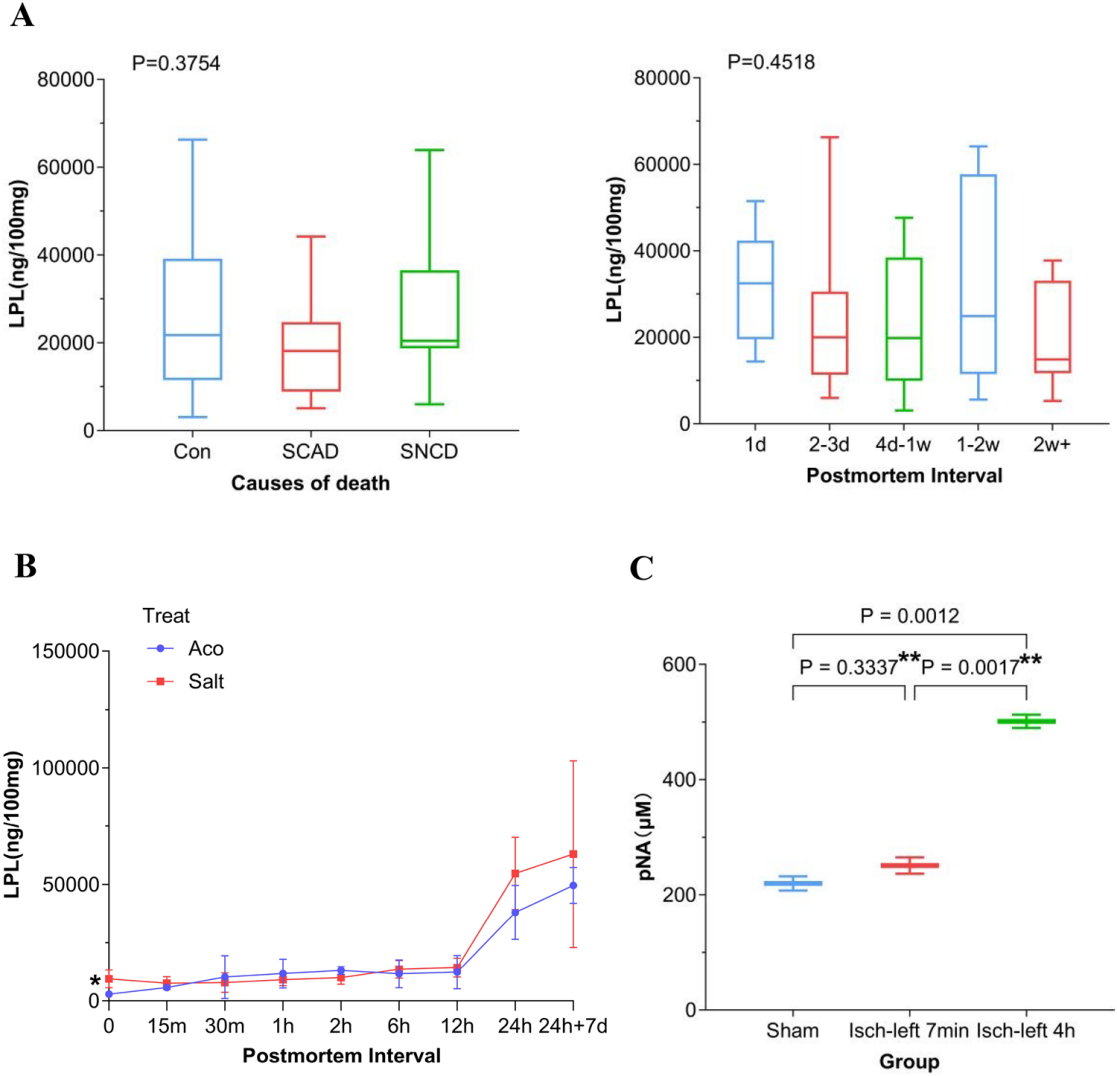
LPLs levels in human (**A**) and rat (**B**) postmortem ventricular myocardia. C, the activity of caspase3 in ischemic myocardia, indicating cell death. *SCAD* sudden death caused by coronary artery disease, *SNCD* sudden death caused by heart disease except for coronary artery disease, *Con* all-cause except for heart disease, *d* day, *w* week, *m* minute, *h* hour. In (**B**), 24 h + 7 d indicates that the corpses were preserved at room temperature for 24 h followed by freezing at −20 ℃ for 7 days, other time points indicate the corpses were preserved at room temperature. *Isch-left* left ventricular ischemia. ANOVA test was used in (**A–C**). *Aco* aconitine. *P < 0.01.

Given the decreasing of LPLs level in the ventricular myocardium in acute myocardial ischemia-caused SCD and drug-mimic SUD in rat, we speculated the LPLs might serve as biomarkers of SCD. Sixty-six specimens of ventricular myocardia from forensic cases were enrolled in this study. Unfortunately, we found no significant difference in LPLs level in human ventricular myocardia between three groups of causes of death: (1) coronary artery disease, (2) heart diseases except for coronary artery disease and (3) all-cause except for heart disease (*P* = 0.38, Fig. 3A). We further tested whether the level was different between different postmortem intervals. The result was negative compared between from postmortem intervals of one day to more than 2 weeks (*P* = 0.45, Fig. 3A). We speculated that postmortem change was responsible for the results of human specimens. So we tested the LPLs level in postmortem rat ventricular myocardium. In the ventricular myocardium isolated immediately after asystole from rats of aconitine-simulated SUD, the LPLs level was significantly lower than that in control group (*P* < 0.01, Fig. 3B), consistent with the result in Supplementary Fig. S6. However, from no longer than postmortem 15 min on, the statistic difference between SUD and control groups was absent, and the accumulating of LPLs became much faster from postmortem 12 h on. Such an postmortem accumulation of LPLs hindered them from serving as applicable biomarkers of SCD for forensic purpose. Given that LPLs accumulated in long time electric shock (Fig. 2B), ischemia (Fig. 2C) and postmortem interval (Fig. 3B), we speculated that the LPLs accumulation was correlated with cell death. We found the activation of caspase 3 in ventricular myocardia was significantly up-regulated when ischemia lasted 4 h (*P* = 0.0012), but not when lasted 7 min (*P* = 0.33) (Fig. 3C). Taken together, these results suggested that LPLs accumulation may be one of the results of cell death caused by various reasons, but not a specific mediator leading to arrhythmias in acute myocardial ischemia.

## Discussion

The causes of SUD is difficult to determine due to comprehensively negative results in postmortem examination^3,4,6,7,9^. Biomarkers with specific changes in SUD attack may function in forensic test, and will spill a light into understanding its molecular mechanism. Here we investigated the lipidomic profiles in the ventricular myocardia of SUD, and found LPLs level decreased in III° atrioventricular block and ventricular fibrillation (Table 1, Fig. 2A and Supplementary Fig. S6). Unfortunately, postmortem accumulation of LPLs annihilated their potential value as usable biomarkers in forensic examination.

Surprisingly, results in this study contradicted the previous conclusion that LPLs conferred arrhythmogenesis during acute myocardial infarction. In previous studies, LPLs, technically it should be LPCs, accumulated in the myocardium immediately after the onset of myocardial ischemia^10–15^, and resulted in arrhythmias^10,16,17^, as well as cardiac damage, in vitro^15,18^.

In this study, onset of ischemia and other arrhythmia-inducing factors caused decrease of LPLs initially. The decrease kept on for no less than 26 min in acute myocardial ischemia (Fig. 2C, Table S3), and for longer than 20 s in electric shock (Fig. 2B, Table S2). In one of the previous studies, electric shock for 20 s after two-minute ischemia resulted in decrease of LPCs in both ischemic and non-ischemic myocardia^12^, consistent with our result. But the authors had not explained the inconsistency of results between electric shock and ischemia in their study. What was more important, our experiments showed that arrhythmias in acute myocardial ischemia were elicited before the accumulation of LPLs. Moreover, LPLs accumulation caused by four-hour ischemia was independent of high risk of arrhythmias. These in vivo results indicated that arrhythmias induced by myocardial ischemia was not mediated by LPLs accumulation. The LPLs accumulation owing to four-hour myocardial ischemia was accompanied by cell death. From postmortem 12 h on, LPLs increased dramatically. These results suggested that LPLs accumulation probably be a result, rather than a reason of cardiac damage. LPCs was reported to mediate the efferocytosis of apoptotic cardiomyocytes by resident macrophages and thus play roles in inflammation inhibition and myocardial repair after ischemic injury^19,20^.

Our results indicated that LPLs decrease was not dependent on the onset of arrhythmias. However, whether LPLs decrease mediate the onset of arrhythmias remains to be elucidated. The U-turn change of LPLs level suggested that LPLs may be versatile in the regulations of different stages during myocardial infarction.

With exogenous addition of LPLs, multiple mechanisms under which LPLs mediate arrhythmias were discovered^21–31^. However, it may need to reassess whether they are truth in vivo. Some studies used LPC-induced myocardial injury as a model to study various anti-injury methods^32–35^. This study alerted us this model is worthy of caution because it may not be consistent with the actual situation in vivo, reducing the practical significance of the anti-injury methods.

LPLs accumulation in ischemic myocardium was reported to be the result of glycerophospholipidolysis mediated by Ca^2+^-independent phospholipase A2^36^. But other studies reported the LPLs accumulation was due to reduced decomposing but not enhanced synthesis^37^, independent of the Ca^2+^-independent phospholipase A2^38^. In this study, we did not observe persuasive synchronous changes of metabolites up- or down-stream of LPLs. So, further studies are needed to eliminate the chaos in this point.

In conclusion, in this study we observed a U-turn change of LPLs level in acute myocardial ischemia, contradicting the viewpoint that LPLs accumulate in the myocardium and confer arrhythmogenesis during acute myocardial infarction. It is time to reassess the roles of LPLs in myocardial infarction and SCD.

### Supplementary Information

Supplementary Information 1.

Supplementary Information 2.

## Data Availability

The datasets used and analyzed during the current study are available from the corresponding author on a reasonable request.
