# Supplementary material for: Arrhythmia onsets triggered by acute myocardial ischemia are not mediated by lysophosphoglycerides accumulation in ventricular myocardium
Source: Sci Rep. 2024 Apr 26;14:9589. doi: 10.1038/s41598-024-57047-5 (PMC11053080; doi:10.1038/s41598-024-57047-5)
Supplement: Supplementary file 2 — Supplementary Information 2. [file 41598_2024_57047_MOESM2_ESM.docx]

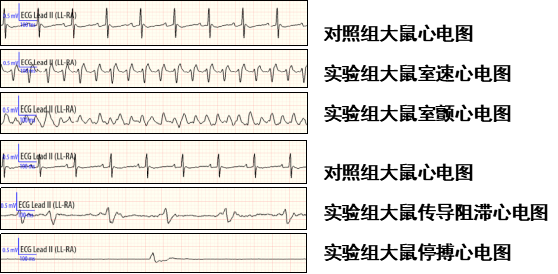


normal ECG

ventricular tachycardia

ventricular fibrillation

atrioventricular block

asystole


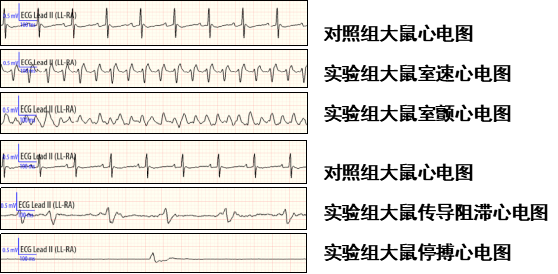


Fig. S1. Electrocardiograms in rats.

**A**

**B**

**C**

Fig. S2. Base peak spectrograms of QC samples in cation (A) and anion (B) modes, and in PCA score diagram (C).


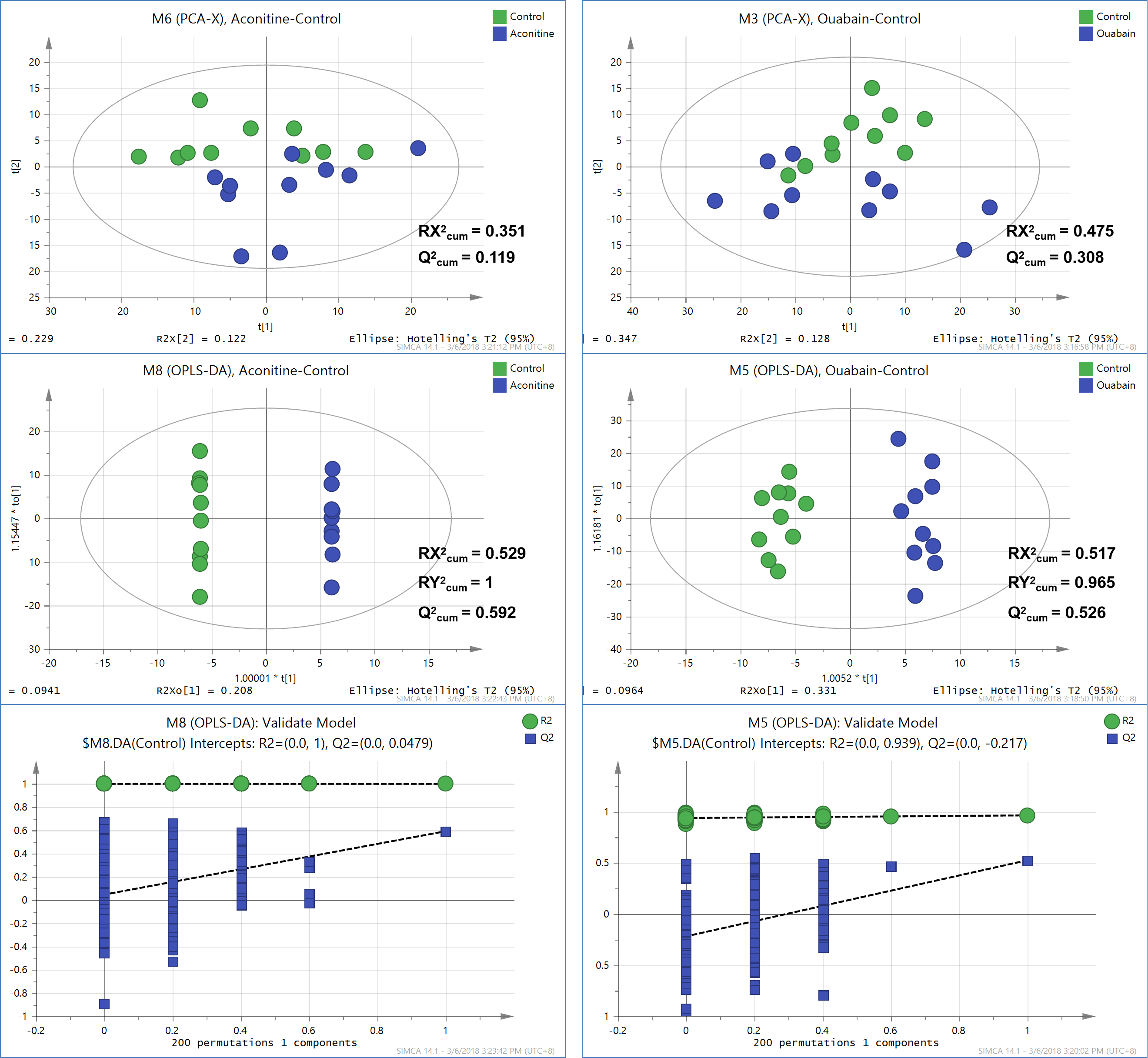


Fig. S3. PCA score plots based on lipid profiles (top), OPLS-DA score plots (middle) and permutation test plots (bottom) of OPLS-DA based on cation lipid profiles. The subscript cum indicates cumulative parameters of model evaluation from 7-fold cross-validation.


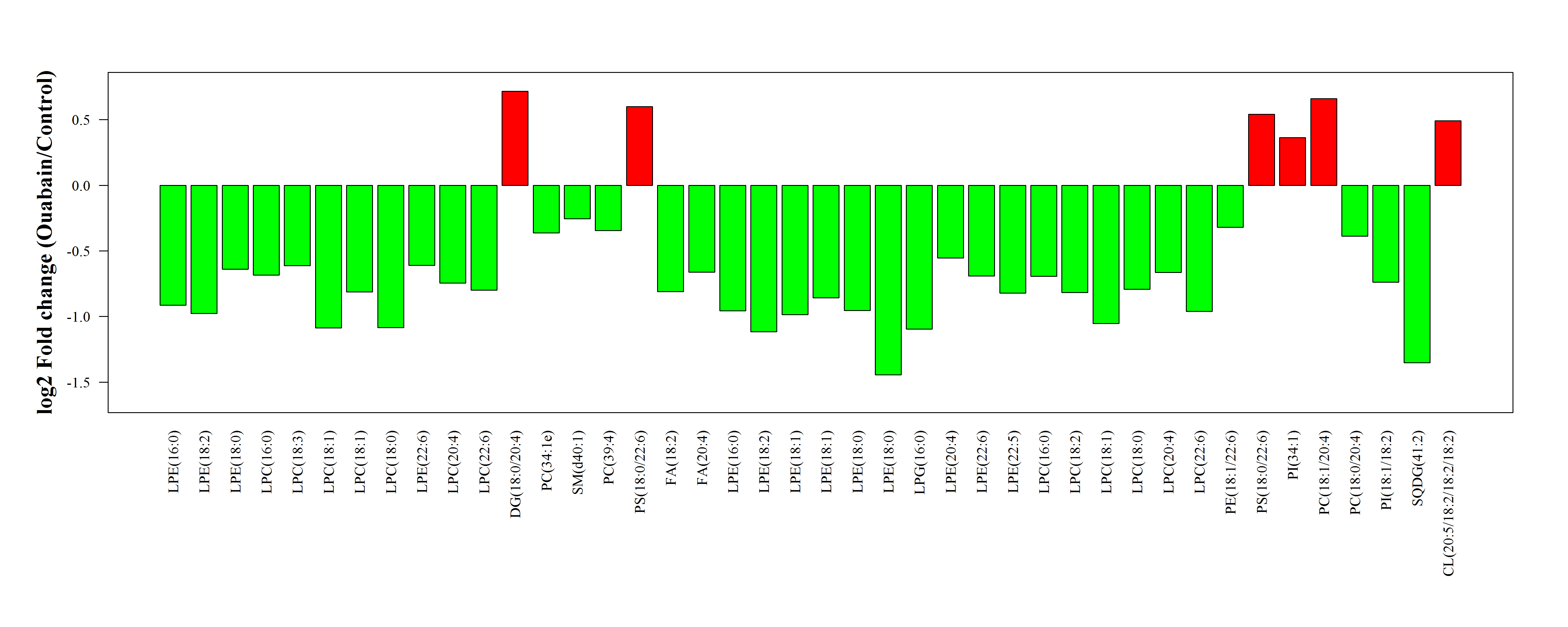

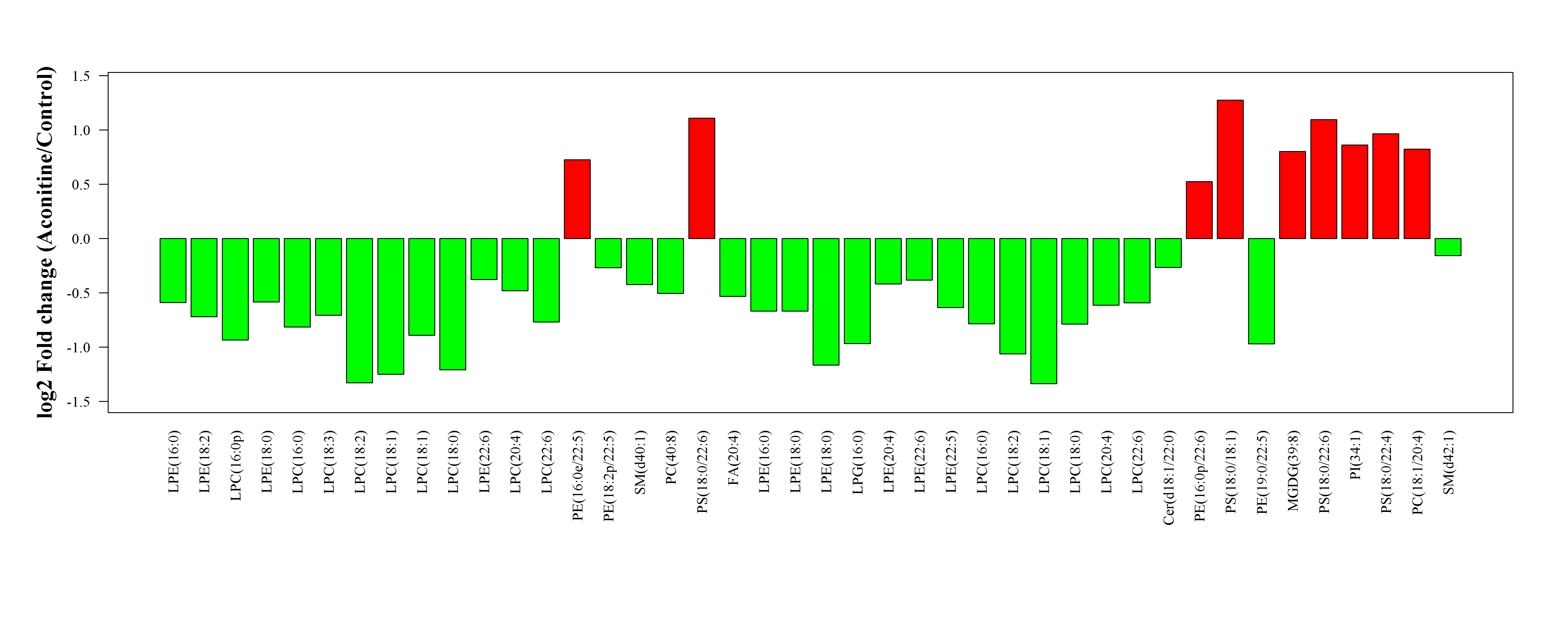


Fig. S4. Lipid ions screened out on the criteria of *p*<0.05 and VIP>1 in aconitine- and ouabain-simulated SUD models. N=10 in each of the groups.

Fig. S5. Hierarchical clustering heatmap based on the differential lipid ions common in aconitine- and ouabain-simulated SUD models. Aco: aconitine, Oua: ouabain, n=10 in each of the groups.


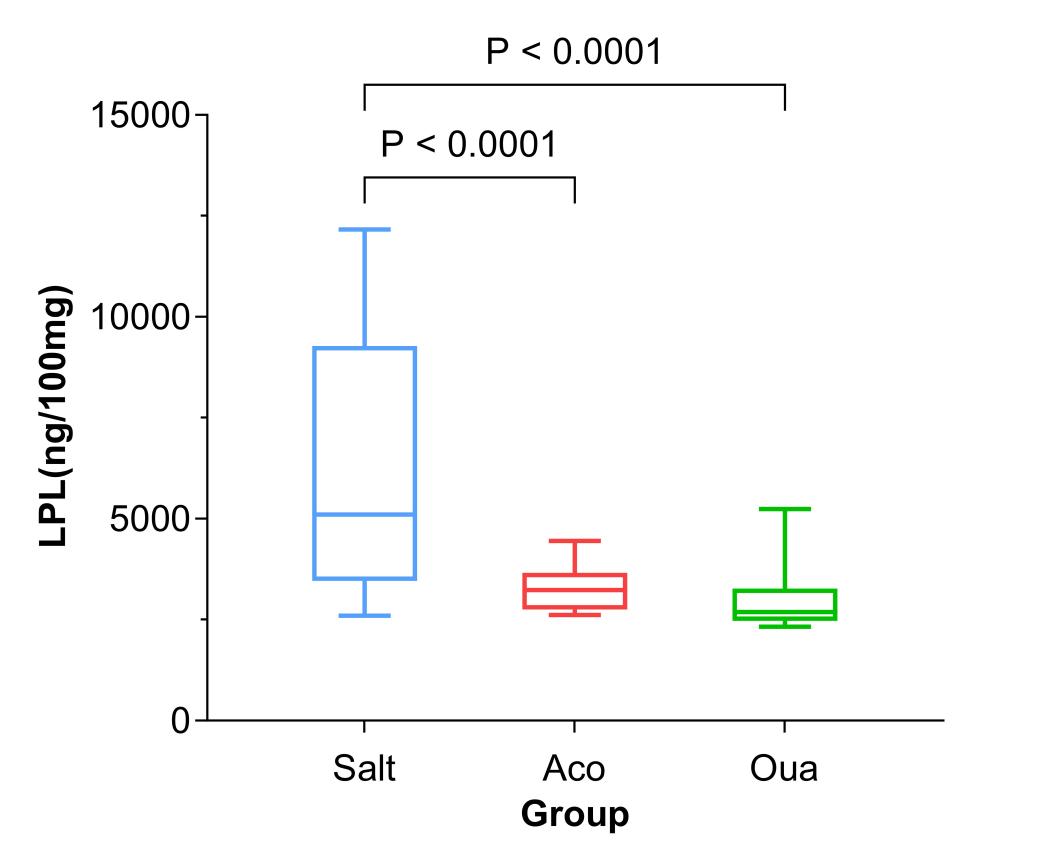


Fig. S6. LPLs levels in ventricular myocardia in aconitine- and ouabain-simulated SUD models. Aco: aconitine, Oua: ouabain, n=20 in each group.

Table S1 LPLs levels in the whole ventricular myocardium of ouabain-induced atrioventricular block

| Lipid | Oua-Ⅰ°Ⅱ° vs Salt | | Oua-Ⅲ°30s vs Salt | | Oua-Ⅲ°>30s vs Salt | |
| --- | --- | --- | --- | --- | --- | --- |
|  | FC | *P* | FC | *P* | FC | *P* |
| LPLs | 0.61 | 0.35 | 0.24 | 0.02 | 0.39 | 0.11 |

FC: Fold Change, LPLs: the total content of 7 lysophospholipids

Table S2 LPLs levels in whole-ventricular myocardia of electric shock with 220V voltage

| Group | LPLs | |
| --- | --- | --- |
|  | FC | *P* |
| EI-10s vs Control | 0.55 | 0.1868 |
| EI-20s vs Control | 0.36 | 0.0352 |
| EI-40s vs Control | 0.48 | 0.1067 |
| EI-2m vs Control | 0.79 | 0.8004 |
| EI-5m vs Control | 2.27 | 0.0008 |
| EI’-10s vs Control | 0.23 | 0.0050 |
| EI’-20s vs Control | 0.33 | 0.0141 |
| EI’-40s vs Control | 0.23 | 0.0051 |
| EI’-2m vs Control | 0.54 | 0.1258 |

EI: left upper-right lower limb current path, EI’: lower-lower limbs current path.

Table S3 LPLs levels in the ventricular myocardia of acute left myocardial ischemia

| Group | LPLs | | Ischemia time (minute) | Arrhythmia time (minute) |
| --- | --- | --- | --- | --- |
|  | FC | *P* |  |  |
| left ventricular myocardia | | | | |
| Isch-30s vs Sham | 0.24 | 0.0018 | 0.5 | 0 |
| Isch-3m vs Sham | 0.31 | 0.0148 | 3 | 0 |
| Isch-7m vs Sham | 0.33 | 0.0036 | 7 | 0 |
| Isch-10m vs Sham | 0.48 | 0.1821 | 10 | 0 |
| VT-1.5m vs Sham | 0.28 | 0.0014 | 8.3 | 2.3 |
| AVB-15s vs Sham | 0.39 | 0.0095 | 1.3 | 0.6 |
| AVB>30s vs Sham | 0.40 | 0.0118 | 8.4 | 6.2 |
| VF-15s vs Sham | 0.32 | 0.0028 | 5 | 1.3 |
| VF>30s vs Sham | 0.66 | 0.5579 | 9 | 5.3 |
| Rec1 vs Sham | 0.36 | 0.0140 | 26 | 0.7 |
| Rec2 vs Sham | 0.55 | 0.3590 | 26 | 0.7 |
| Isch-4h vs Sham | 1.20 | 0.0078 | 240 | 6 |
| Isch-right vs Sham | 0.43 | 0.0792 | 3 | 0 |
| right ventricular myocardia | | | | |
| Isch-30s vs Sham | 1.26 | ＞0.9999 | 0.5 | 0 |
| Isch-3m vs Sham | 1.24 | ＞0.9999 | 3 | 0 |
| Isch-7m vs Sham | 0.65 | 0.9942 | 7 | 0 |
| Isch-10m vs Sham | 0.48 | ＞0.9999 | 10 | 0 |
| VT-1.5m vs Sham | 0.28 | 0.9956 | 8.3 | 2.3 |
| AVB-15s vs Sham | 0.39 | ＞0.9999 | 1.3 | 0.6 |
| AVB>30s vs Sham | 0.40 | 0.9890 | 8.4 | 6.2 |
| VF-15s vs Sham | 0.32 | ＞0.9999 | 5 | 1.3 |
| VF>30s vs Sham | 0.66 | ＞0.9999 | 9 | 5.3 |
| Rec1 vs Sham | 0.36 | ＞0.9999 | 26 | 0.7 |
| Rec2 vs Sham | 0.55 | 0.9987 | 26 | 0.7 |
| Isch-right vs Sham | 0.30 | 0.0030 | 3 | 0 |

AVB: Ⅲ° atrioventricular block, Isch: ischemia, VT: ventricular tachycardia, AVB: Ⅲ° atrioventricular block, VF: ventricular fibrillation, Rec 1: the ECG recovered to normal following transient Ⅰ°-Ⅱ° atrioventricular block; Rec 2: the ECG recovered to normal following transient Ⅲ° atrioventricular block.
